# Equilibrium of Global Amphibian Species Distributions with Climate

**DOI:** 10.1371/journal.pone.0034420

**Published:** 2012-04-12

**Authors:** Mariana Munguía, Carsten Rahbek, Thiago F. Rangel, Jose Alexandre F. Diniz-Filho, Miguel B. Araújo

**Affiliations:** 1 Departamento de Biodiversidad y Biología Evolutiva, Museo Nacional de Ciencias Naturales, CSIC, Madrid, Spain; 2 Departamento de Zoología, Instituto de Biología, Universidad Nacional Autónoma de México, Distrito Federal, México; 3 Department of Biology, Center for Macroecology, Evolution and Climate, University of Copenhagen, Copenhagen, Denmark; 4 Departamento de Ecologia, ICB, Universidade Federal de Goiás, Goiânia, GO, Brazil; 5 Cátedra Rui Nabeiro em Biodiversidade, CIBIO, Universidade de Évora, Évora, Portugal; CNRS, University of Montpellier II, France

## Abstract

A common assumption in bioclimatic envelope modeling is that species distributions are in equilibrium with contemporary climate. A number of studies have measured departures from equilibrium in species distributions in particular regions, but such investigations were never carried out for a complete lineage across its entire distribution. We measure departures of equilibrium with contemporary climate for the distributions of the world amphibian species. Specifically, we fitted bioclimatic envelopes for 5544 species using three presence-only models. We then measured the proportion of the modeled envelope that is currently occupied by the species, as a metric of equilibrium of species distributions with climate. The assumption was that the greater the difference between modeled bioclimatic envelope and the occupied distribution, the greater the likelihood that species distribution would not be at equilibrium with contemporary climate. On average, amphibians occupied 30% to 57% of their potential distributions. Although patterns differed across regions, there were no significant differences among lineages. Species in the Neotropic, Afrotropics, Indo-Malay, and Palaearctic occupied a smaller proportion of their potential distributions than species in the Nearctic, Madagascar, and Australasia. We acknowledge that our models underestimate non equilibrium, and discuss potential reasons for the observed patterns. From a modeling perspective our results support the view that at global scale bioclimatic envelope models might perform similarly across lineages but differently across regions.

## Introduction

A common assumption underlying several large-scale ecological models is that species distributions are in equilibrium with contemporary climate; in other words, that species are generally present in climatically suitable areas while being absent from unsuitable ones [1]. Obviously, this construct is a simplification because species distributions are rarely, if ever, in full equilibrium with contemporary climate. The question is how far species distributions are from climatic equilibrium and, more specifically, how equilibrium varies across taxa and regions. Addressing these questions is not only of theoretical interest. It is also important for understanding the limits to predicting climate change impacts on biodiversity [2], [3]. Even though the assumption of equilibrium underpins all models that empirically estimate species–climate relationships, only a few studies have quantified the departure of observed distributions from potential ones. Existing studies were restricted to Europe [1], [4] and Mexico [5]. In the case of the European analyses, equilibrium was estimated using a small proportion of the total extent of species distributions, thus leading to an underestimation of the realized niches.

Another study that overcomes the circularity of quantifying species-climate equilibrium using range filling of potential distributions (which themselves are constrained by biotic interactions and dispersal limitation), used physiologically-derived estimates of the fundamental niche for a small number of bird species in North and South America, and compared them with estimates of the realized niche [6]. Unfortunately, such approach is unfeasible for all amphibians of the world.

Generally, studies investigating species-climate equilibrium with correlative approaches found high levels of non equilibrium, particularly among species with poor dispersal abilities [7]. However, given the small number of studies addressing this question, it is difficult to generalize. Questions such as ‘are patterns of non-equilibrium geographically or taxonomically structured?’ remain unanswered. Furthermore, any bias introduced by measuring degrees of equilibrium using incomplete species distributions has not been quantified.

Here, we seek to contribute to this debate by providing the first global analysis of equilibrium patterns for an entire class of organisms. We estimated climate envelopes for the world amphibian species using familiar bioclimatic envelope techniques. We then measured the proportion of each species' potential climatic distribution that is currently occupied. The underlying assumption of our test is that the greater the difference between potential and occupied distributions, the greater the likelihood that species distributions would not be at equilibrium with contemporary climate [4]. We then explored how equilibrium of species distributions varies across taxa and regions.

## Methods

### Data

Distributions of 5544 amphibian species were extracted from the Global Amphibian Assessment database (IUCN 2004). Polygons of species ranges were resampled at a 2-degree latitude-longitude grid cell resolution. Species that occurred in two (*N* = 235) or more biogeographical regions (*N* = 7) were not modeled to ensure comparability between the global and regional analysis (see description below). Restricted range sized species are known to cause statistical problems for fitting of bioclimatic envelope models [8]–[10]. We quantified this problem and found that the median range size of amphibians of the world at a 2-degree resolution is equal to three pixels. So, imposing a rule of exclusion for restricted range species would drastically limit the number of species that could be modeled. To deal with the problem, we split the species data by range sizes and analyzed results for sets of species with >0 cells (N_Global_ = 5544, N_Regional_ = 5309), >5 cells (N_Global_ = 2005, N_Regional_ = 1816), >10 cells (N_Global_ = 1321, N_Regional_ = 1163), and >15 cells (N_Global_ = 1021, N_Regional_ = 886). Although the quality of the models for the data sets including the rarest species is reduced, we assumed that if the patterns emerging are qualitatively similar across the different subsets of species, then the conclusions should be relatively insensitive to the problem of modeling species with restricted ranges. Bioclimatic envelope models were then fitted for the amphibian species using five climate variables selected among those previously reported to be important for hylids (tree frogs) [11] and salamanders [12]: (1) the minimum temperature of the coldest month; (2) the maximum temperature of the warmest month; (3) the annual mean temperature; (4) annual precipitation; and (5) temperature seasonality (standard deviation * 100). Climate data were extracted from the WorldClim database [13].

### Climate envelopes

In order to assess inter-model variability [14]–[16], species potential climatic distributions were calculated with BIOCLIM [17], Euclidian (ED), and Mahalanobis distances [18], using a combination of climate variables and observed species occurrences. BIOCLIM estimates species envelopes by defining the bounding hyper-box that encloses all records of the species in the climatic space. To characterize the hyper-box, it creates a rectilinear envelope in the climatic space, defined by the most extreme records of the species on each axis. To minimize the effect of outliers, species records are sorted along each variable, and the records that lie within a certain percentile range of the data are used for characterizing the envelopes. In this study, we applied a percentile range of 95%, the default option in most studies using this approach [19]. BIOCLIM tends to overestimate species potential distributions slightly more than other presence-only models [20] and significantly more than presence/absence methods [21]. This overestimation of observed ranges leads to an inflation of false positives (i.e., a species predicted to occur where it has not been recorded), a tendency that contributes to the low-ranking of BIOCLIM when compared with methods that fit more-complex response curves and that adjust projections to balance false positives and false negatives equally. However, if the purpose of the model is to estimate the climatic envelope, then BIOCLIM is potentially as good as many of the concurrent methods available [4].

Euclidian and Mahalanobis distances are conceptually similar to BIOCLIM, but instead of generating a squared hyper-volume, they define circular or elliptical shapes in climatic hyperspace. The idea is to measure the similarity of each occurrence to the mean (or centre) of the ecological space. In Euclidian distances, the distance (D_E_) between each occurrence, or grid cell, to the species' centroid is given by:
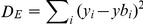
Where y_i_ is the value of the i-th environmental variable and yb_i_ is the mean of the variable. For the Mahalanobis distance, the distance D_M_ is given by
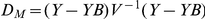
Where **Y** is the vector containing the values of the environmental variables in a cell and **YB** is the mean vector across all cells, and **V** is the covariance matrix among these variables. Thus, geometrically, whereas BIOCLIM defines the surface range envelope in environmental space as a square (or rectangle), the distances will allow circles, in the case of Euclidian distances (assuming independence effects of the variables) or ellipses in the case of Mahalanobis distances (taking into account the correlation among variables).

Only BIOCLIM was able to characterise climate envelopes for species with <15 records of occurrence. The full set of analysis included: >0B (species with at least 1 record using BIOCLIM), >5B, >10B, >15B, >15MD (from Mahalanobis), and >15ED (from Euclidian Distance). The options for parameterisation of these two methods were the same as defined for BIOCLIM. All models were implemented with BIOENSEMBLES [22], [23] software for computer intensive ensemble forecasting.

### Equilibrium

**Figure 1:**
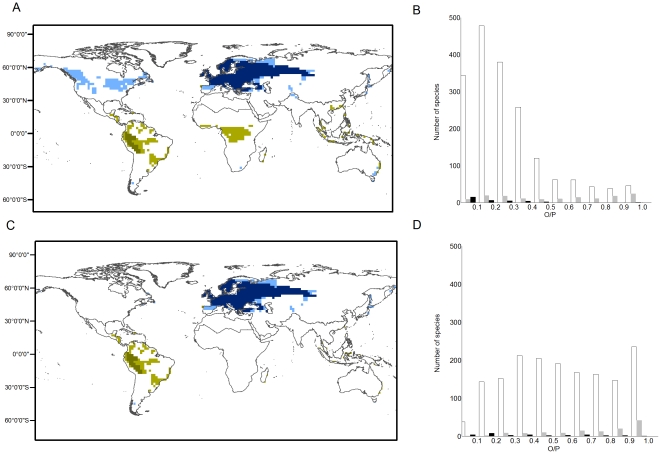
Observed and modeled potential distributions for two selected species: *Lissotriton vulgaris* (Salamandridae, Caudata) in the Palearctic region (Blue) and *Chiasmocleis ventrimaculata* (Microhylidae, Anura) (Green) in the Neotropic region. Dark colors are observed distributions and light colors are potential distributions; the smaller the difference between observed and potential distributions, the greater the expected equilibrium of species with climate. (A) Global analysis, in which models calibrated for species occurring in a particular biogeographical region are allowed to project climate space globally; (C) regional analysis, in which models are not allowed to project climate space beyond the biogeographical region in which the species occurs. Frequency distribution of equilibrium values (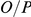) at the (B) global and (D) regional scales. White bars represent Anura, shaded represent Caudata, and black represent Gymnophiona.

For each species, we calculated the potential climate envelope (*P*) and compared it with its observed distribution (*O*) (Figure 1). The ratio between *O* and *P* 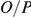 was interpreted as a measure of the equilibrium of species distributions with contemporary climate (see also [4], [5]); measurements of 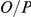 values closer to 1 were considered to approach equilibrium. We then calculated the mean geographic position (*GP*) of each species' centroid by matrix multiplication: 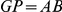, where ***A*** was a transposed matrix of species presence/absence within each grid cell and ***B*** was a matrix with latitude and longitude coordinates for grid cells [24]. The degree of equilibrium of species distributions with climate 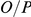 was then associated with each species' ***GP*** and compared across space and taxa. Kruskal-Wallis tests (i.e., a non-parametric test identical to one-way analysis of variance with the data replaced by ranks) were used to test the equality of median 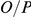 values between groups.

### Global and Regional analysis

Projections of climatic envelopes were initially made for the entire world, but a regional analysis was also obtained by masking out climate envelopes occurring outside the biogeographical region where the species occurs (Figure 1). The global analysis was expected to provide quantification of the degree of global equilibrium of species distributions, i.e., discounting for the effects of limited dispersal across biogeographical regions and providing an estimate expected to be closer to the ‘abiotically suitable area’ available for the species [25]. In contrast, the regional analysis accounted for cross-regional dispersal limitation and other biome-level biotic contingencies [26], thus providing a more rigorous estimate of the potential distribution of species that implicitly accounts for the effects of dispersal and biotic interactions in reducing the abiotically suitable area for the species (Figure 1). Biogeographical regions, or biomes, were classified following the divisions of Sclater [27] and Wallace [28], later renamed by Olson *et al.* [29]: Nearctic, Palaearctic, Indo-Malay, Australasia, Afrotropics, and Neotropic. We added an additional region, Madagascar, because it is now widely accepted that this region holds a markedly distinct and more diverse biota than anticipated, particularly among amphibian species [30] (Table 1).

**Table 1:** Geography, richness and equilibrium descriptions across biogeographical regions.

	Afrotropic(without islands)	Australasia(without islands)	Indo-Malay (without islands)	Madagascar	Nearctic(without islands)	Neotropic(without islands)	Palearctic(without islands)
Area (number of 2decimal degrees cells)	513	488	187	28	1212	559	2041
Maximum and Minimum Latitude	21° HN35° HS	3° HN47° HS	33°HN3° HN	11° HS25° HS	83°HN21°HN	27°HN55°HS	81°HN 17°HN
Number of total latitude geographic coordinates	56°	50°	30°	14°	62°	77°	64°
Percentage in Tropic- Subtropic/Temperate regions	100/0	96.5/3.5	100/0	100/0	30.6/69.4	80.5/19.5	35.9/64.1
Longitudinal wider extent	17°W51°E	113°E179°E	67°E21°E	41°E51°E	179°W13°W	109°W35°W	17°W179°E
Number of longitude geographic coordinates	68	66	54	10	166	74	196
Number of Biomes	9	9	10	5	11	12	10
Species richness (% total amphibians)	686 (12.9)	516 (9.7)	661 (12.4)	218 (4.1)	249(4.7)	2684 (50.6)	295(5.6)
O/P	0.55	0.70	0.48	0.89	0.83	0.45	0.55

For both the global and the regional analyses, comparisons of 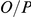 were made across biogeographical realms and taxonomic groupings at the level of Order: Anura (frogs and toads), Caudata (newts and salamanders), and Gymnophiona (caecilians). The regional comparison was necessary to tease apart signals that might arise because of the different biogeographical histories of the regions. The taxonomic comparison was undertaken to investigate whether the ecological properties of the groups affected their levels of equilibrium with contemporary climate. Differences in 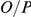 values in regional and global analyses were compared with U-Mann Whithney, which is a non-parametric test of whether two independent samples of observations have equally large values [31]. Results of the analysis are reported for species with >5 cells (>5B), since they are qualitatively similar to the patterns obtained with species with broader ranges (>10B and 15B) and among different bioclimatic models (15B, 15MH and 15ED; see full set of results in Table S1, S2 and Figure S1).

## Results

**Figure 2:**
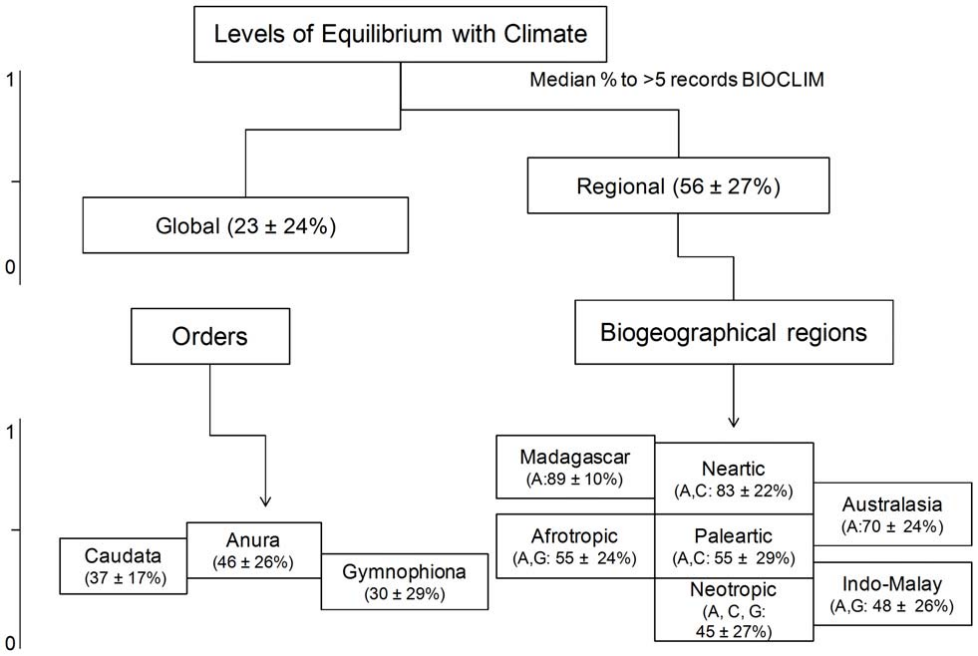
Diagram of climate equilibrium levels at global and regional scales, biogeographical regions and orders. We only show Neotropical orders in the diagram because that show all orders: Caudata (C = salamanders), Anura (A = frogs and toads) and Gymnophiona (G = cecilians), however all regions were analyzed by region (see text).

We found 1) significant differences in equilibrium (i.e., 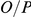) among species both when analysis were made including the global potential distributions of the species, which is an attempt to estimate abiotically suitable area for them, and when potential distributions were restricted to the biogeographical region where the species occurs, thus accounting for limited dispersal preventing cross-continent colonization for most amphibians; 2) higher equilibrium among amphibian faunas in Madagascar, Nearctic and Australasian regions compared to the faunas in the Neotropic, Indo-Malayan, Afrotropics, and Palaearctic regions; and 3) that equilibrium values were not significantly different among amphibian orders (Figure 2).

### Global and Regional analysis

Amphibians occupy 30% (Median = 23%) of their potential distribution at global scale. The frequency distribution of equilibrium (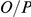; Figure 1) value is positive skewed (1.34), i.e., most species tend towards low 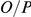 values, and displays positive *kurtosis* (1.19; Table 2), i.e., with heavy tail and an overly peaked with regards to a normal distribution. In contrast, amphibians were found to occupy 57% of their potential range when analysis were performed at regional scale (Median = 56%; Table 2). The frequency distribution of the equilibrium (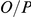; Figure 1) value is slightly skewed to the right (positive skewness = 0.024) and peaked (low kurtosis = −1.11; Table 2). The frequency distributions of equilibrium values in the global vs. regional analysis were significantly different (U-Mann Whithney = 8098757; z = 29.68, N = 1816, P<0.001). Orders showed greater median values of 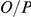 in the regional analysis when compared with the global analysis. This outcome is to be expected because the global analysis involves an inflation of the estimated distribution.

**Table 2:** Descriptive statistics of the level of equilibrium among world.

Scale	Biogeographical region	Order	Mean	Median	Standard deviation	Skewness	Kurtosis	N
Global		All Orders	0.30	0.23	0.24	1.34	1.19	2005
Regional		All Orders	0.57	0.56	0.27	0.02	−1.11	1816
	Neotropic	All Orders	0.48	0.45	0.27	0.26	−0.97	729
		Anura	0.48	0.46	0.27	0.25	−0.97	695
		Caudata	0.33	0.37	0.17	−0.31	−1.47	8
		Gymnophiona	0.38	0.30	0.29	0.67	−1.01	27
	Palearctic	All Orders	0.58	0.55	0.29	0.03	−1.32	138
		Anura	0.56	0.50	0.29	0.09	−1.27	97
		Caudata	0.64	0.63	0.28	−0.08	−1.51	41
	Nearctic	All Orders	0.78	0.83	0.22	−0.83	−0.31	137
		Anura	0.75	0.79	0.24	−0.63	−0.89	57
		Caudata	0.81	0.83	0.20	−0.95	0.29	80
	Afrotropic	All Orders	0.55	0.55	0.24	0.11	−0.80	328
		Anura	0.56	0.55	0.24	0.10	−0.81	325
		Gymnophiona	0.43	0.54	0.23	−1.68	--------	3
	Indo-Malay	All Orders	0.55	0.48	0.26	0.57	−0.86	229
		Anura	0.55	0.49	0.27	0.56	−0.88	226
		Gymnophiona	0.45	0.42	0.15	0.87	--------	3
	Madagascar	Anura	0.88	0.89	0.1	−0.88	1.07	72
	Australasia	Anura	0.69	0.70	0.24	−0.24	−1.11	183

Results with the >5B dataset were generally consistent with >10B, >15B, >15MH and >15ED, and are shown in the supplementary material. But the data with >0 records showed discrepant results, particular regarding the frequency distribution of 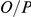 values, which were negatively skewed for the full set of species (see Table S1). In contrast a positively skewed for the subsets of species with larger range sizes and among different bioclimatic models (>5B, >10B, and 15B, 15MH and 15ED; see Table S1) except 15B at regional scale, but the value was almost zero (15B skewness = −0.03). The interpretation of the results for the full set of species (>0B) is therefore driven by the smallest range size species for which models provide less reliable projections of the potential distribution of species.

The greater difference among bioclimatic models was observed in ED model which showed the lowest values of equilibrium because they showed larger P areas than the other models. However, the relative difference among regions and orders was similar to those observed for the other bioclimatic models.

### Regional differences

**Figure 3:**
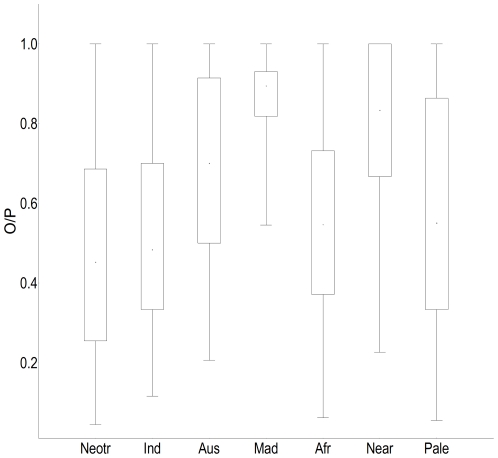
Degree of climatic equilibrium for amphibians within the seven biogeographical regions. Boxes are the percentiles from 25 to 75% around 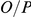 medians, lines indicate the standard deviation.

**Figure 4:**
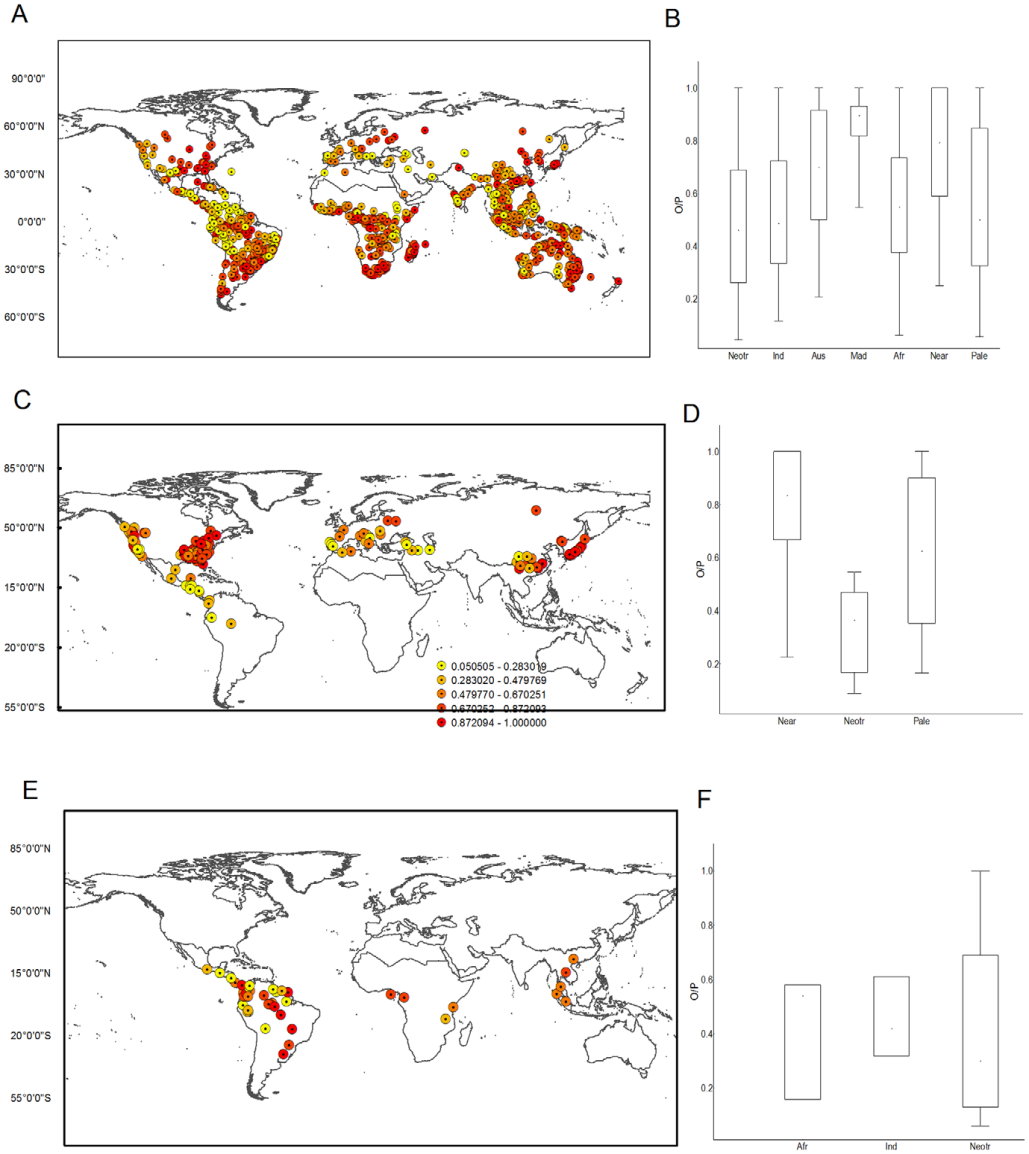
Distribution of the centroids of species geographical distributions and their respective level of equilibrium (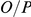): (A) Anura, (C) Caudata, and (E) Gymnophiona. Differences among regions in each order (B) Anura (N = 1655) (D) Caudata (N = 129) (F) Gymnphiona (N = 33).

In the Nearctic, Madagascar and Australasian regions amphibian species showed significantly higher equilibrium with climate (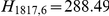, P<0.0001; Median = 83.88 and 70% respectively) than amphibians inhabiting the other regions (Median = Neotropic 45%, Paleartic 55%, Afrotropics 55%, Indo-Malay 48%; Figure 3, Table 1, see Table S1, S2 and Figure S1). It is noteworthy that amphibians in the Nearctic show higher levels of O/P than the climatically similar Palaearctic. When looking at the results by Order, similar patterns emerge. Unsurprisingly, Anurans showed a similar pattern to all amphibians combined as they represent the majority of amphibians (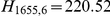, P<0.0001; Figure 3, Figure 4, Table 3, see Figure S1). But Caudata only occurs in three biogeographical regions, and showed the same patters as observed with Anuran, i.e., greater equilibrium in the Nearctic, followed by the Palaearctic and the Neotropic (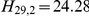, P<0.0001; Figure 4). In contrast, equilibrium values for Gymnophiona, were not significantly significant across regions (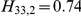, P = 0.69; Figure 4).

**Table 3:** Degree of climatic equilibrium for amphibians within the seven biogeographical regions.

	Afrotropic	Australasian	Indo-Malay	Madagascar	Nearctic	Neotropics	Palearctics
Afrotropic	____	5.33***	0.54NS	8.95***	8.07***	4.17**	0.78NS
Australasian		____	5.43***	4.84***	2.91NS	9.31***	3.66*
Indo-Malay			_____	8.97***	8.03***	3.05*	1.17NS
Madagascar				_____	2.36NS	11.68***	7.47***
Neartic					_____	11.80***	6.15***
Neotropics						______	3.85*
Paleartics							_____

*** *P*<0.0001,

** *P*<0.001,

* *P*<0.05,

NS = Non significant.

Kruskal Wallis test between pairs of regions differences.

### Taxonomic differences

The three amphibian Orders did not show significant differences in equilibrium within regions (P>0.01). Because not all orders are present in every region, we compared Anura vs. Caudata in the Nearctic (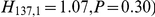 and in the Palaearctic (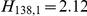, P = 0.15) and the Anura vs. Gymnophiona in the Afrotropics (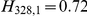, P = 0.4) and in the Indo-Malay region (here Gymnophiona showed only 2 species with more than 5 cells, so we did not report the results). Anura is the only order present in Madagascar and Australasia. Finally were compared the three orders in the Neotropic (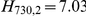, P = 0.03).

## Discussion

The proportion of the estimated climate envelopes of species that are currently occupied does not differ significantly among amphibian orders. In contrast, significant differences were found among biogeographical regions. Overall, amphibians occupied between 30%–57% (global versus regional analysis) of their potential distributions. Differences between equilibrium at global versus regional levels, highlight the importance of carefully considering the appropriate scale of analysis [25]. Nevertheless, the range of values in our study match those recorded elsewhere with other organisms. For example, Svenning & Skov [4] found that a sample of European temperate tree species occupied 38.3% of their potential distributions, whereas Munguía *et al.* [5] found that this proportion was about 50% for Mexican mammals. Finding the appropriate geographical extent for analysis is not trivial, but we generally recommend that in studies using the 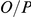 ratio as a measure of range filling or equilibrium, the minimum study area should be as large as the largest geographical range of species in the analysis to control for the geographical range [5]. In practice, this strategy involves running the analysis using coherent biogeographical units, with common evolutionary histories, such as the regions used herein.

Fundamentally, tough, the levels of range filling among amphibian species are typically low. Our measurement of equilibrium is probably inflated because we measure equilibrium as range filling of potential distributions rather than that of abiotically suitable areas or fundamental niches, which is the quantity of interest. The true level of equilibrium is thus likely to be lower than estimated. Nevertheless, measured low equilibrium among amphibians is unsurprising since the species in the group have generally low dispersal abilities, are often being unable to track suitable climate as it changes through time [32]. However, significant differences in the degree of range filling among regions indicate that the ability of species to track climate changes varies regionally. According to our models, amphibians in the Nearctic, Madagascar, and Australasia have greater levels of equilibrium with contemporary climate than amphibians in the Neotropic, Afrotropics, Indo-Malay, and the Palaearctic. It follows, that the ability to model species distributions, particularly when models are used for transferability [33], [34] or extrapolation [35], is greatest in the regions where species have higher levels of equilibrium with climate.

Our study, being based on correlations and on a rather coarse resolution data for species distributions and climate, does not illuminate as to the reasons why range filling varies among amphibians in different parts of the world. Speculations can be offered and some might provide inspiration for future studies. For example, it is noteworthy that two of the biogeographical regions with higher equilibrium are also among the smaller, i.e., Madagascar and Australasia. Just because these regions are small, compared to biogeographical regions that span across vast continents, it is more likely that species inhabiting them can colonize a greater proportion of suitable areas. Another region that is small but has amphibian faunas with low equilibrium with climate is the Indo-Malay region (Table 2). However, this region comprises an archipelago, so dispersal into suitable sites in unoccupied islands is very unlikely.

Another noteworthy pattern is the difference in equilibrium between amphibian species in the Nearctic and the Palaearctic. The former has much higher levels of equilibrium than the latter. Both regions are large and both are exposed to temperate conditions with marked seasonality. Species being exposed to such climate conditions are expected to have evolved thermoregulatory strategies that facilitate adaptation to a wider range of conditions than, for example, tropical species [36]–[39]. Wider tolerances to climate favor, all other things being equal, dispersal. Several authors have noted that post-glacial colonization in the Palaearctic and the Nearctic were different and that such differences might explain why Quaternary extinctions were greater in the western Palaearctic than in the Nearctic [40]. To put it simply, the argument goes that the longitudinal orientation of mountain ranges in Europe prevented effective colonization of southern refugia (and back) of some species, while the latitudinal orientation of the major mountains in north American acted as continental-wide corridor easing dispersal [41], [42].

Another possibility to explain differences between equilibrium patterns between the Nearctic and Palaearctic is that the extent and position of deserts in Palaearctic could act as strong physical barriers to dispersal. Amphibians require water or humidity to live and reproduce and they cannot disperse through wide arid lands; estimates are that 37% of Caudata are strictly aquatic, whereas the figure is 75% for Anurans [43]. Deserts occupy 10.4% of the Palaearctic and they are generally present in the central and the southern fringes of the region. So, they are likely to play an important role as barriers. In contrast, deserts in Nearctic are in the south-west and account for only 3% of the region.

The description of patterns of equilibrium in species distributions with climate is just beginning. Understanding of the mechanisms determining the geographical variation in equilibrium is still limited. Our study provides the first description of such patterns, for an entire clade of organisms across their global distribution. Alternative studies with other groups, with different ecologies and dispersal abilities, and with data at different spatial scales of resolution, will help provide a broader and more complete picture. Progress will also require that inferences about equilibrium with bioclimatic models are compared with other approaches, such that provided with eco-physiologically driven measurements of species niches [44], [45] that allow comparisons between species observed distributions versus the abiotically suitable areas or fundamental niche (instead of the provided comparison with species potential distributions or realized niches) [6]. The latter approach is not practical when analyzing large number of species for which eco-physiological data is unavailable and alternatives might involve running and macroecological analysis of diversity and assemblage composition against contemporary climate [1], [32], [41], [46]. Improved understanding of how and how much species tracked past climate changes, and how they occupy current suitable climates is critical to understand and forecast the potential responses of species to ongoing climate changes.

## Supporting Information

Figure S1 **Degree of equilibrium of climate for amphibians at regional scale in the seven biogeographical regions.** >0B set of species with O equal to more or equal than 1 cell, >10B more than 10 cells, >15B more than 15 cells using BIOCLIM, >15MD more than 15 using Mahalanobis, and >15ED more than 15 using Euclidian Distance. Boxes are the percentiles from 25 to 75% around O/P medians, and lines indicate the standard deviation. (A) All orders; (B) Anura; (C) Caudata; (D) Gymnophiona. Neotropic (Neotr), Indo-Malay (Ind), Australasia (Aus), Madagascar (Mad), Afrotropic (Afr), Nearctic (Near), Palaearctic (Pale).
(TIF)

Table S1 **Descriptive statistics of the level of equilibrium among the amphibian species at global and regional scales.**
(DOCX)

Table S2 **Test of the differences in the degree of climatic equilibrium between pairs of regions.** The upper-right diagonal shows the consistency of the Kruskal Wallis tests among different bioclimate models (first position, BIOCLIM: 15B/second position, Mahalanobis distance:15MD/third position, Euclidian distance:15ED). The lower-left diagonal shows differences between different geographical ranges (first position, >0B records/second position, >10B/third position, >15B). Biogeographical regions: Afrotropic (Af), Australasia (Aus), Indo-Malay (Ind), Madagascar (Mad), Nearctic (Near), Neotropic (Neotr), Palaearctic (Palear).
(DOCX)
