# Supplementary figures and images for: Equilibrium of Global Amphibian Species Distributions with Climate
Source: PLoS One. 2012 Apr 12;7(4):e34420. doi: 10.1371/journal.pone.0034420 (PMC3325238; doi:10.1371/journal.pone.0034420)

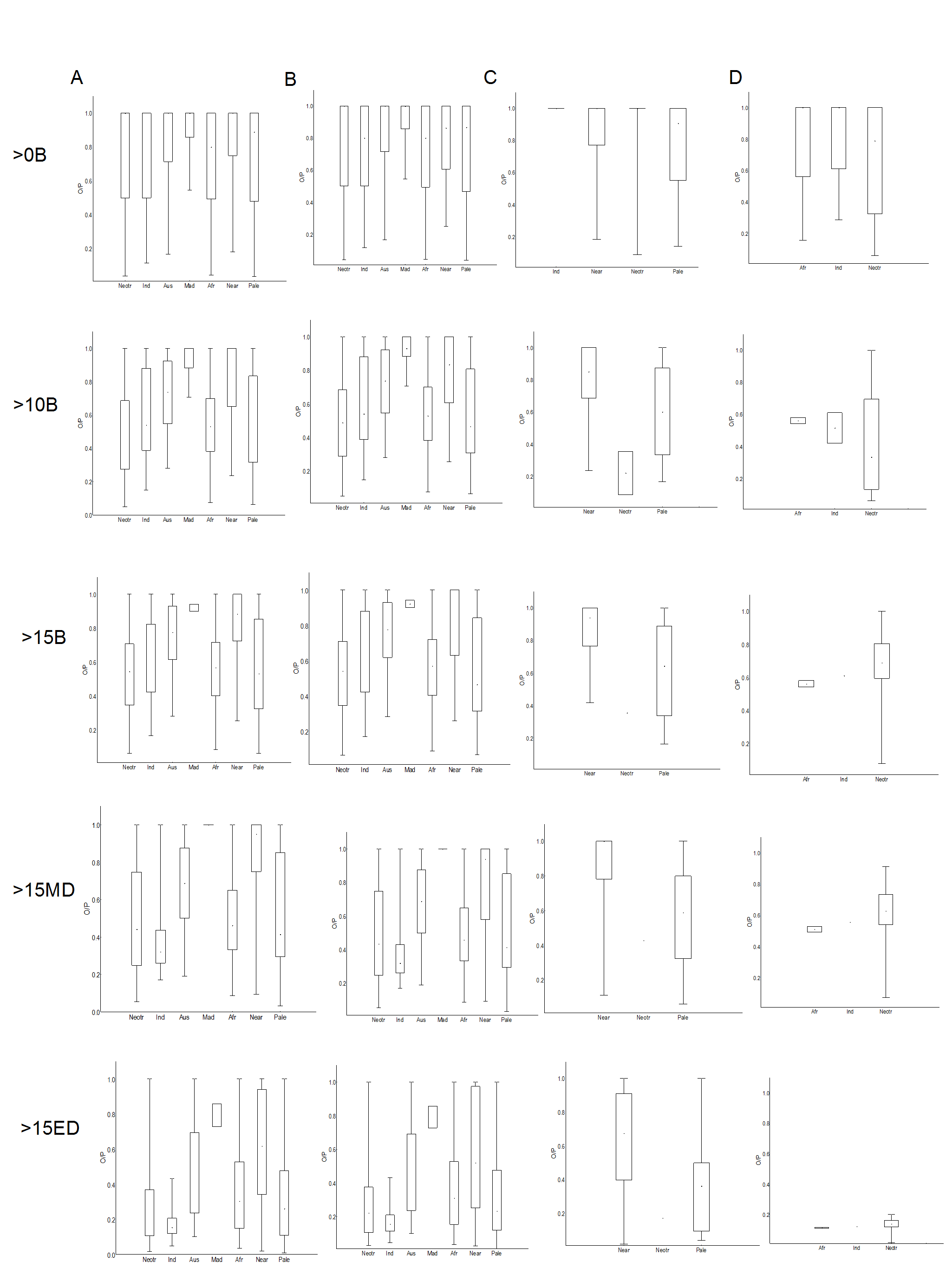

Supplement: Figure S1 — Degree of equilibrium of climate for amphibians at regional scale in the seven biogeographical regions. >0B set of species with O equal to more or equal than 1 cell, >10B more than 10 cells, >15B more than 15 cells using BIOCLIM, >15MD more than 15 using Mahalanobis, and >15ED more than 15 using Euclidian Distance. Boxes are the percentiles from 25 to 75% around O/P medians, and lines indicate the standard deviation. (A) All orders; (B) Anura; (C) Caudata; (D) Gymnophiona. Neotropic (Neotr), Indo-Malay (Ind), Australasia (Aus), Madagascar (Mad), Afrotropic (Afr), Nearctic (Near), Palaearctic (Pale). (TIF) [file pone.0034420.s001.tif]
