# Supplementary material for: Equilibrium of Global Amphibian Species Distributions with Climate
Source: PLoS One. 2012 Apr 12;7(4):e34420. doi: 10.1371/journal.pone.0034420 (PMC3325238; doi:10.1371/journal.pone.0034420)
Supplement: Table S1 — Descriptive statistics of the level of equilibrium among the amphibian species at global and regional scales. (DOCX) [file pone.0034420.s002.docx]

| **Scale** | **Biogeographic region** | **Order** | **Models** | **Mean** | **Median** | **Standard deviation** | **Skewness** | **Kurtosis** | **N** |
| --- | --- | --- | --- | --- | --- | --- | --- | --- | --- |
| Global |  | All Orders | >0B | 0.61 | 0.66 | 0.37 | -0.19 | -1.68 | 5544 |
|  |  |  | >10B | 0.28 | 0.23 | 0.21 | 1.42 | 1.80 | 1321 |
|  |  |  | >15B | 0.30 | 0.25 | 0.21 | 1.46 | 1.95 | 1021 |
|  |  |  | >15MH | 0.24 | 0.18 | 0.19 | 1.34 | 1.55 | 1021 |
|  |  |  | >15ED | 0.16 | 0.06 | 0.24 | 2.47 | 5.36 | 1021 |
| Regional |  | All Orders | >0B | 0.77 | 1.00 | 0.29 | -0.91 | -0.58 | 5309 |
|  |  |  | >10B | 0.57 | 0.56 | 0.26 | 0.05 | -0.99 | 1163 |
|  |  |  | >15B | 0.60 | 0.60 | 0.25 | -0.03 | -0-95 | 886 |
|  |  |  | >15MH | 0.53 | 0.48 | 0.28 | 0.27 | -1.16 | 886 |
|  |  |  | >15ED | 0.33 | 0.25 | 0.27 | 1.09 | 0.31 | 886 |
|  | Neotropic | All Orders | >0B | 0.76 | 1.00 | 0.31 | -0.87 | -0.73 | 2684 |
|  |  |  | >10B | 0.49 | 0.49 | 0.25 | 0.17 | -0.91 | 459 |
|  |  |  | >15B | 0.54 | 0.55 | 0.24 | 0.03 | -0.81 | 334 |
|  |  |  | >15MH | 0.50 | 0.44 | 0.29 | 0.32 | -1.13 | 334 |
|  |  |  | >15ED | 0.27 | 0.21 | 0.23 | 1.58 | 2.59 | 334 |
|  |  | Anura | >0B | 0.75 | 1.00 | 0.31 | -0.83 | -0.81 | 2396 |
|  |  |  | >10B | 0.49 | 0.49 | 0.25 | 0.17 | -0.89 | 442 |
|  |  |  | >15B | 0.53 | 0.54 | 0.24 | 0.05 | -0.81 | 324 |
|  |  |  | >15MD | 0.49 | 0.43 | 0.29 | 0.34 | -1.14 | 324 |
|  |  |  | >15ED | 0.27 | 0.22 | 0.23 | 1.54 | 2.44 | 324 |
|  |  | Caudata | >0 | 0.88 | 1.00 | 0.24 | -1.78 | 1.80 | 212 |
|  |  |  | >10 | 0.22 | 0.22 | -------- | -------- | -------- | 2 |
|  |  |  | >15B | 0.36 | 0.36 | -------- | -------- | -------- | 1 |
|  |  |  | >15MH | 0.43 | 0.43 | -------- | -------- | -------- | 1 |
|  |  |  | >15ED | 0.17 | 0.17 | -------- | -------- | -------- | 1 |
|  |  | Gymnophiona | >0B | 0.68 | 0.79 | 0.35 | -0.53 | -1.36 | 76 |
|  |  |  | >10B | 0.43 | 0.33 | 0.33 | 0.34 | -1.56 | 15 |
|  |  |  | >15B | 0.63 | 0.69 | 0.27 | -0.93 | 0.95 | 9 |
|  |  |  | >15MH | 0.57 | 0.63 | 0.25 | -0.93 | 0.955 | 9 |
|  |  |  | >15ED | 0.13 | 0.14 | 0.05 | -0.93 | 0.95 | 9 |
|  | Palearctic | All Orders | >0B | 0.74 | 0.89 | 0.30 | -0.76 | -0.86 | 295 |
|  |  |  | >10B | 0.54 | 0.48 | 0.28 | 0.19 | -1.28 | 102 |
|  |  |  | >15B | 0.57 | 0.53 | 0.29 | 0.02 | -1.34 | 73 |
|  |  |  | >15MD | 0.53 | 0.41 | 0.31 | 0.27 | -1.37 | 73 |
|  |  |  | >15ED | 0.34 | 0.29 | 0.26 | 0.77 | -0.22 | 73 |
|  |  | Anura | >0B | 0.73 | 0.87 | 0.31 | -0.73 | -0.90 | 208 |
|  |  |  | >10B | 0.53 | 0.47 | 0.29 | 0.21 | -1.26 | 77 |
|  |  |  | >15B | 0.54 | 0.46 | 0.29 | 0.13 | -1.31 | 46 |
|  |  |  | >15MH | 0.52 | 0.41 | 0.31 | 0.35 | -1.35 | 46 |
|  |  |  | >15ED | 0.33 | 0.23 | 0.27 | 0.81 | -0.26 | 46 |
|  |  | Caudata | >0B | 0.77 | 0.91 | 0.29 | -0.84 | -0.80 | 87 |
|  |  |  | >10B | 0.58 | 0.60 | 0.27 | 0.17 | -1.42 | 22 |
|  |  |  | >15B | 0.64 | 0.64 | 0.27 | -0.33 | -1.28 | 17 |
|  |  |  | >15MH | 0.56 | 0.59 | 0.33 | 0.06 | -1.37 | 17 |
|  |  |  | >15ED | 0.34 | 0.36 | 0.28 | 0.84 | 0.36 | 17 |
|  | Nearctic | All Orders | >0B | 0.85 | 1.00 | 0.21 | -1.30 | 0.79 | 249 |
|  |  |  | >10B | 0.79 | 0.84 | 0.22 | -0.83 | -0.48 | 98 |
|  |  |  | >15B | 0.82 | 0.88 | 0.21 | -1.04 | -0.07 | 85 |
|  |  |  | >15MH | 0.80 | 0.95 | 0.30 | -1.46 | 0.42 | 85 |
|  |  |  | >15ED | 0.57 | 0.62 | 0.34 | -0.25 | -1.24 | 85 |
|  |  | Anura | >0B | 0.78 | 0.86 | 0.24 | -0.78 | -0.76 | 75 |
|  |  |  | >10B | 0.77 | 0.83 | 0.24 | -0.71 | -0.87 | 50 |
|  |  |  | >15B | 0.79 | 0.87 | 0.24 | -0.88 | -0.57 | 46 |
|  |  |  | >15MH | 0.77 | 0.94 | 0.32 | -1.19 | -0.25 | 46 |
|  |  |  | >15ED | 0.55 | 0.52 | 0.35 | -0.05 | -1.41 | 46 |
|  |  | Caudata | >0B | 0.87 | 1.00 | 0.18 | -1.57 | 2.14 | 174 |
|  |  |  | >10B | 0.81 | 0.8 | 0.20 | -0.97 | 0.10 | 48 |
|  |  |  | >15B | 0.85 | 0.94 | 0.18 | -1.08 | 0.09 | 39 |
|  |  |  | >15MH | 0.83 | 1.00 | 0.28 | -1.77 | 1.89 | 39 |
|  |  |  | >15ED | 0.60 | 0.67 | 0.34 | -0.53 | -0.87 | 39 |
|  | Afrotropic | All Orders | >0B | 0.73 | 0.80 | 0.29 | -0.57 | -1.06 | 686 |
|  |  |  | >10B | 0.55 | 0.53 | 0.21 | 0.16 | -0.57 | 243 |
|  |  |  | >15B | 0.57 | 0.57 | 0.20 | 0.22 | -0.69 | 204 |
|  |  |  | >15MH | 0.50 | 0.46 | 0.21 | 0.55 | -0.44 | 204 |
|  |  |  | >15ED | 0.37 | 0.30 | 0.26 | 0.79 | -0.31 | 204 |
|  |  | Anura | >0B | 0.72 | 0.80 | 0.29 | -0.56 | -1.06 | 666 |
|  |  |  | >10B | 0.55 | 0.53 | 0.21 | 0.16 | -0.59 | 241 |
|  |  |  | >15B | 0.57 | 0.57 | 0.20 | 0.22 | -0.71 | 202 |
|  |  |  | >15MH | 0.50 | 0.46 | 0.21 | 0.55 | -0.46 | 202 |
|  |  |  | >15ED | 0.37 | 0.30 | 0.26 | 0.78 | -0.32 | 202 |
|  |  | Gymnophiona | >0B | 0.81 | 1.00 | 0.30 | -1.23 | -0.11 | 20 |
|  |  |  | >10B | 0.56 | 0.56 | -------- | -------- | -------- | 2 |
|  |  |  | >15B | 0.56 | 0.56 | -------- | -------- | -------- | 2 |
|  |  |  | >15MH | 0.11 | 0.51 | -------- | -------- | -------- | 2 |
|  |  |  | >15ED | 0.11 | 0.11 | -------- | -------- | -------- | 2 |
|  | Indo-Malay | All Orders | >0B | 0.75 | 0.86 | 0.28 | -0.54 | -1.23 | 661 |
|  |  |  | >10B | 0.59 | 0.54 | 0.27 | 0.45 | -1.11 | 131 |
|  |  |  | >15B | 0.61 | 0.56 | 0.25 | 0.46 | -1.05 | 96 |
|  |  |  | >15MH | 0.37 | 0.32 | 0.16 | 1.45 | 2.44 | 96 |
|  |  |  | >15ED | 0.17 | 0.16 | 0.08 | 1.20 | 1.26 | 96 |
|  |  | Anura | >0B | 0.74 | 0.80 | 0.28 | -0.49 | -1.28 | 613 |
|  |  |  | >10B | 0.59 | 0.54 | 0.27 | 0.44 | -1.14 | 129 |
|  |  |  | >15B | 0.61 | 0.56 | 0.25 | 0.45 | -1.07 | 95 |
|  |  |  | >15MH | 0.37 | 0.32 | 0.16 | 1.50 | 2.62 | 95 |
|  |  |  | >15ED | 0.17 | 0.16 | 0.08 | 1.16 | 1.19 | 95 |
|  |  | Gymnophiona | >0B | 0.83 | 1.00 | 0.24 | -0.93 | -0.67 | 39 |
|  |  |  | >10B | 0.51 | 0.51 | -------- | -------- | -------- | 2 |
|  |  |  | >15B | 0.61 | 0.61 | -------- | -------- | -------- | 1 |
|  |  |  | >15MH | 0.55 | 0.55 | -------- | -------- | -------- | 1 |
|  |  |  | >15ED | 0.12 | 0.12 | -------- | -------- | -------- | 1 |
|  | Madagascar | Anura | >0B | 0.91 | 1.00 | 0.13 | -1.32 | 0.50 | 218 |
|  |  |  | >10B | 0.91 | 0.93 | 0.10 | -1.05 | 0.22 | 18 |
|  |  |  | >15B | 0.91 | 0.92 | -------- | -------- | -------- | 2 |
|  |  |  | >15MH | 1.00 | 1.00 | -------- | -------- | -------- | 2 |
|  |  |  | >15ED | 0.79 | 0.79 | -------- | -------- | -------- | 2 |
|  | Australasia | Anura | >0B | 0.85 | 1.00 | 0.22 | -1.29 | 0.49 | 516 |
|  |  |  | >10B | 0.72 | 0.74 | 0.21 | -0.28 | -1.10 | 112 |
|  |  |  | >15B | 0.75 | 0.77 | 0.20 | -0.48 | -0.86 | 92 |
|  |  |  | >15MH | 0.67 | 0.69 | 0.22 | -0.21 | -1.02 | 92 |
|  |  |  | >15ED | 0.49 | 0.42 | 0.29 | 0.58 | -0.96 | 92 |
