# Supplementary material for: Equilibrium of Global Amphibian Species Distributions with Climate
Source: PLoS One. 2012 Apr 12;7(4):e34420. doi: 10.1371/journal.pone.0034420 (PMC3325238; doi:10.1371/journal.pone.0034420)
Supplement: Table S2 — Test of the differences in the degree of climatic equilibrium between pairs of regions. The upper-right diagonal shows the consistency of the Kruskal Wallis tests among different bioclimate models (first position, BIOCLIM: 15B/second position, Mahalanobis distance:15MD/third position, Euclidian distance:15ED). The lower-left diagonal shows differences between different geographical ranges (first position, >0B records/second position, >10B/third position, >15B). Biogeographical regions: Afrotropic (Af), Australasia (Aus), Indo-Malay (Ind), Madagascar (Mad), Nearctic (Near), Neotropic (Neotr), Palaearctic (Palear). (DOCX) [file pone.0034420.s003.docx]

|  | Afrotropic | Australasian | Indomalayan | Madagascar | Nearctic | Neotropics | Palearctics |
| --- | --- | --- | --- | --- | --- | --- | --- |
| Af. | ------------ | ***/***/* | NS/NS/*** | NS/*/NS | ***/***/ NS | NS / NS /* | NS / NS / NS |
| Aus. | ***/***/*** | ------------ | ***/ NS /*** | NS / NS / NS | NS / NS / NS | ***/***/*** | ***/*/** |
| Ind. | NS/NS/* | ***/*/ *** | ------------ | NS /***/*** | ***/***/*** | NS / NS / NS | NS / NS / NS |
| Mad. | ***/***/NS | NS/NS/ NS | ***/***/NS | ------------ | NS / NS / NS | NS /*/* | NS /*/ NS |
| Near. | **/***/*** | NS/NS/ NS | NS/***/*** | NS/NS/NS | ------------ | ***/***/*** | ***/***/NS |
| Neotr. | */NS/NS | */***/*** | NS/NS/NS | */***/NS | NS/***/*** | ------------ | NS / NS / NS |
| Palear. | */NS/NS | **/ ***/*** | NS/NS/NS | ****/***/NS | NS/***/*** | NS/NS/NS | ------------ |
| ****P*<0.0001, ***P*<0.001, **P*<0.05, NS=Non significant | | | | | | | |
